# Epigenetic dysregulation of autophagy in sepsis-induced acute kidney injury: the underlying mechanisms for renoprotection

**DOI:** 10.3389/fimmu.2023.1180866

**Published:** 2023-05-05

**Authors:** Shankun Zhao, Jian Liao, Maolei Shen, Xin Li, Mei Wu

**Affiliations:** ^1^ Department of Urology, Taizhou Central Hospital (Taizhou University Hospital), Taizho, Zhejiang, China; ^2^ Department of Nephrology, Jiaxing Hospital of Traditional Chinese Medicine, Jiaxing, Zhejiang, China; ^3^ Educational Administration Department, Chongqing University Cancer Hospital, Chongqing, China

**Keywords:** sepsis, acute kidney injury, autophagy, protection, mechanism

## Abstract

Sepsis-induced acute kidney injury (SI-AKI), a common critically ill, represents one of the leading causes of global death. Emerging evidence reveals autophagy as a pivotal modulator of SI-AKI. Autophagy affects the cellular processes of renal lesions, including cell death, inflammation, and immune responses. Herein, we conducted a systematic and comprehensive review on the topic of the proposed roles of autophagy in SI-AKI. Forty-one relevant studies were finally included and further summarized and analyzed. This review revealed that a majority of included studies (24/41, 58.5%) showed an elevation of the autophagy level during SI-AKI, while 22% and 19.5% of the included studies reported an inhibition and an elevation at the early stage but a declination of renal autophagy in SI-AKI, respectively. Multiple intracellular signaling molecules and pathways targeting autophagy (e.g. mTOR, non-coding RNA, Sirtuins family, mitophagy, AMPK, ROS, NF-Kb, and Parkin) involved in the process of SI-AKI, exerting multiple biological effects on the kidney. Multiple treatment modalities (e.g. small molecule inhibitors, temsirolimus, rapamycin, polydatin, ascorbate, recombinant human erythropoietin, stem cells, Procyanidin B2, and dexmedetomidine) have been found to improve renal function, which may be attributed to the elevation of the autophagy level in SI-AKI. Though the exact roles of autophagy in SI-AKI have not been well elucidated, it may be implicated in preventing SI-AKI through various molecular pathways. Targeting the autophagy-associated proteins and pathways may hint towards a new prospective in the treatment of critically ill patients with SI-AKI, but more preclinical studies are still warranted to validate this hypothesis.

## Introduction

Sepsis, one of the common diseases in the intensive care units, seriously threatens the lives of sufferers, contributing to 30-50% of deaths in hospitals (1). As a result of the dysregulated host response to infection, severe systemic inflammation may induce septic shock, disseminated intravascular coagulation (DIC), and progressive multi-organ dysfunction syndrome (MODS) (2). The commonly affected organs include the heart, lungs, liver, brain, intestine, and kidneys. During sepsis, activation of the sympathetic nervous system, the release of vasoactive substances, and endothelial injury, together contribute to the redistribution of blood flow and microcirculation disturbances (3). These factors remarkably damage the kidney tissue (e.g. renal tubular) and therefore induce acute kidney injury (AKI) and even acute renal failure (ARF). ARF was found to be the most common complication of sepsis, accounting for nearly 50% of the incidence rate (4). On the other hand, it was reported that approximately 76% of in-hospital deaths are caused by sepsis-induced AKI (SI-AKI) (5). Since multidrug-resistant bacteria and adverse events are common in sepsis, septic ARF has a significantly higher mortality rate than that of non-sepsis-related ARF (caused by other pathogenic factors or some diseases) (6). To effectively prevent and treat septic AKI or ARF, intensive efforts have been made to develop innovative therapeutic measures and explore the detailed molecular pathological mechanisms underlying SI-AKI. However, due to the complex character of SI-AKI, the exact pathogenesis mechanisms for septic AKI are not completely addressed, prohibiting or arresting effective treatments for septic AKI.

At present, there is growing evidence that autophagy plays a role in the pathogenesis process of SI-AKI (7). Autophagy is an adaptive catabolic process and is commonly correlated to cellular death, protection, or survival. It conserves the degradation of eukaryotic cells and the recycling process, maintaining cellular homeostasis by engulfing cellular targets (i.e., pathogens, unfolded proteins, carbohydrates, lipids, nucleic acids, and damaged organelles) (8). Autophagy can be regulated by a complex signaling network comprised of autophagy-related genes (ATC). Mitophagy and lysophagy are the common types of selective autophagy. Autophagy is considered to be a cellular stress triggered by a multiplicity of adverse environmental cues, i.e., hypoxia, oxidative stress, and nutrient depletion (9). Experimental and clinical findings reveal that autophagy may serve as a pathogenic mediator of human diseases by regulating inflammation, innate immunity, and host defense. Dysregulated or maladaptive autophagy with propathogenic responses was found to be associated with the pathogenesis of disorders (10).

For the kidneys to function normally, autophagy is necessary (11). Besides, the presence of kidney pathologies in mice bearing genetic deletions of key autophagy regulator proteins. For example, in an animal model with targeted deletions of *Atg5* or *Atg7*, chronic kidney disease could be induced (12). Therefore, autophagy may act as a key safeguard against the declination of kidney function. Also, it was reported that autophagy generally protects the kidney from various injuries, e.g. sepsis, renal ischemia-reperfusion (I/R), or exposure to nephrotoxins (13). However, the exact molecular mechanisms underlying the action of autophagy in AKI are still exploring.

In this review, we focused specifically on the roles of autophagy in sepsis-induced AKI according to the current evidence. Elucidating the biological effects of autophagy in septic AKI is of pivotal importance, which may provide strategies and targets for therapeutic interventions in clinical practice.

### A search of the literature

To identify the studies that reported the association between autophagy and sepsis-induced AKI, we conducted a comprehensive literature review in the common-used databases, i.e., MEDLINE, Web of Science, Google Scholar, EMBASE, and Cochrane Library. The reference list in the review or original studies was also retrieved to identify additional relevant studies. Data from relevant studies were extracted using a data collection table. The following information was noted, including the first author of the included studies, year of publication, experimental model or participant, methods for establishing sepsis-induced AKI, the status of autophagy, associated genes or pathways in the action of autophagy, and the main findings of the relevant studies. Finally, there were 41 experimental and clinical studies (7, 14–33) (34–53) included in the review. The selection process for screening the relevant studies shown in **Supplementary Figure 1**. For the methods for establishing the SI-AKI, cecal ligation and puncture (CLP) were used in *in-vivo* studies, while lipopolysaccharide (LPS) was applied in *in vitro* studies.

### Different autophagy levels in septic AKI

Among the 41 relevant studies, 24 of them (24/41, 58.5%) reported that the autophagy status was activated in septic AKI, while eight eligible studies showed that autophagy inhibition occurred in the kidney under sepsis and the remaining nine studies reported that autophagy elevated in early stage but declined in the later phase during sepsis-induced AKI.

Autophagy, a form of programmed cell death different from apoptosis, occurs in all eukaryotic cells and is associated with cellular turnover and energy balance. Death of cells occurs when apoptosis appear**s**, whereas autophagy is a “double-edged sword” for both survival and death (54, 55). Apoptosis and autophagy are interconnected in some way. To protect cells from apoptosis and necrosis under stress, autophagy is commonly activated. Nevertheless, excessive autophagy can increase apoptosis due to mitochondrial damage. Autophagy plays a protective role in proximal tubular cells of the kidney against AKI (11). Of note, autophagy is considered to play a double role during sepsis. Basal autophagy functions the protection of the injuried kidney by eliminating toxic oxidative proteins. However, severe stress (i.e., ROS eruption) may induce excessive autophagy, leading to autophagic cell death (56). It is reported that autophagy is initiated early after sepsis, protecting against endotoxic kidney damage (57). Afterward, autophagic cell death may cause a phase of dysfunction, aggravating the sepsis-induced oxidative injury.

At present, specific molecular mechanisms underlying sepsis-associated AKI are not fully understood. Several potential mechanisms could be involved (58). First, autophagy can protect proximal tubular cells from mitochondrial oxidative stress and ischemic injury. ‘Mitophagy’ is a specific type of autophagy, which is characterized by the clearance of mitochondria by autophagy. Second, autophagy also plays role in protecting proximal tubular cells from DNA damage. Third, autophagy can reduce the abnormal protein accumulation of proximal tubular cells.

Since the level of autophagy flux was different among the 41 included studies, exploring the underlying mechanisms of autophagy is of great interest and has important implications for septic AKI research. Here, we summarize recent laboratory and clinical studies, focusing on critical factors in the pathophysiology of sepsis-associated AKI: microcirculatory dysfunction, inflammation, NOD-like receptor protein 3 inflammasome, microRNAs, extracellular vesicles, autophagy and efferocytosis, inflammatory reflex pathway, vitamin D, and metabolic reprogramming. Lastly, identifying these molecular targets and defining clinical subphenotypes will permit precision approaches in the prevention and treatment of SI-AKI.

#### Autophagy activation in septic AKI

It is possible that autophagy plays a key role in the recovery of AKI by promoting cell survival. Autophagy can be triggered in response to various types of stress, including sepsis. A critical cytoprotective role for autophagy in sepsis-mediated AKI has been found in recent years (11). The protective effects exerted by autophagy may be associated with the removal of damaged mitochondria or mitophagy, which predominantly affects the mitochondria-rich proximal tubule cells (58). Currently, evidence that harnessing the autophagic machinery on SI-AKI is still controversial. The levels of autophagy flux during SI-AKI are different among studies.

Twenty-four publications reported the autophagy level was activated during sepsis-induced AKI. Alexander et al. (27) performed the autopsy on 17 patients who died from coronavirus disease 2019 (COVID-19) and molecular characteristics were compared with archived cases of S-AKI and non-sepsis causes of AKI. They found that the autophagy level was significantly higher in sepsis-induced AKI than in non-sepsis-related AKI (*P*=0.023). However, the autophagy status was comparable between sepsis AKI and COVID AKI (*P*=0.621). This clinical trial indicated that mitochondrial autophagy dysfunction might play a pivotal role in SI-AKI, which might provide novel diagnostic and therapeutic targets for treating SI-AKI. In another clinical study developed by Feng et al. (23), the investigators also revealed the autophagy status was elevated in SI-AKI patients. As a result, exploring the molecular mechanisms underlying the actions of autophagy dysfunction in septic AKI may be clinically instructive. Currently, 24 experimental studies had investigated the association between autophagy and SI-AKI. Nevertheless, the detailed pathomechanisms of autophagy contributed to the pathogenesis of septic AKI.

**Figure 1:**
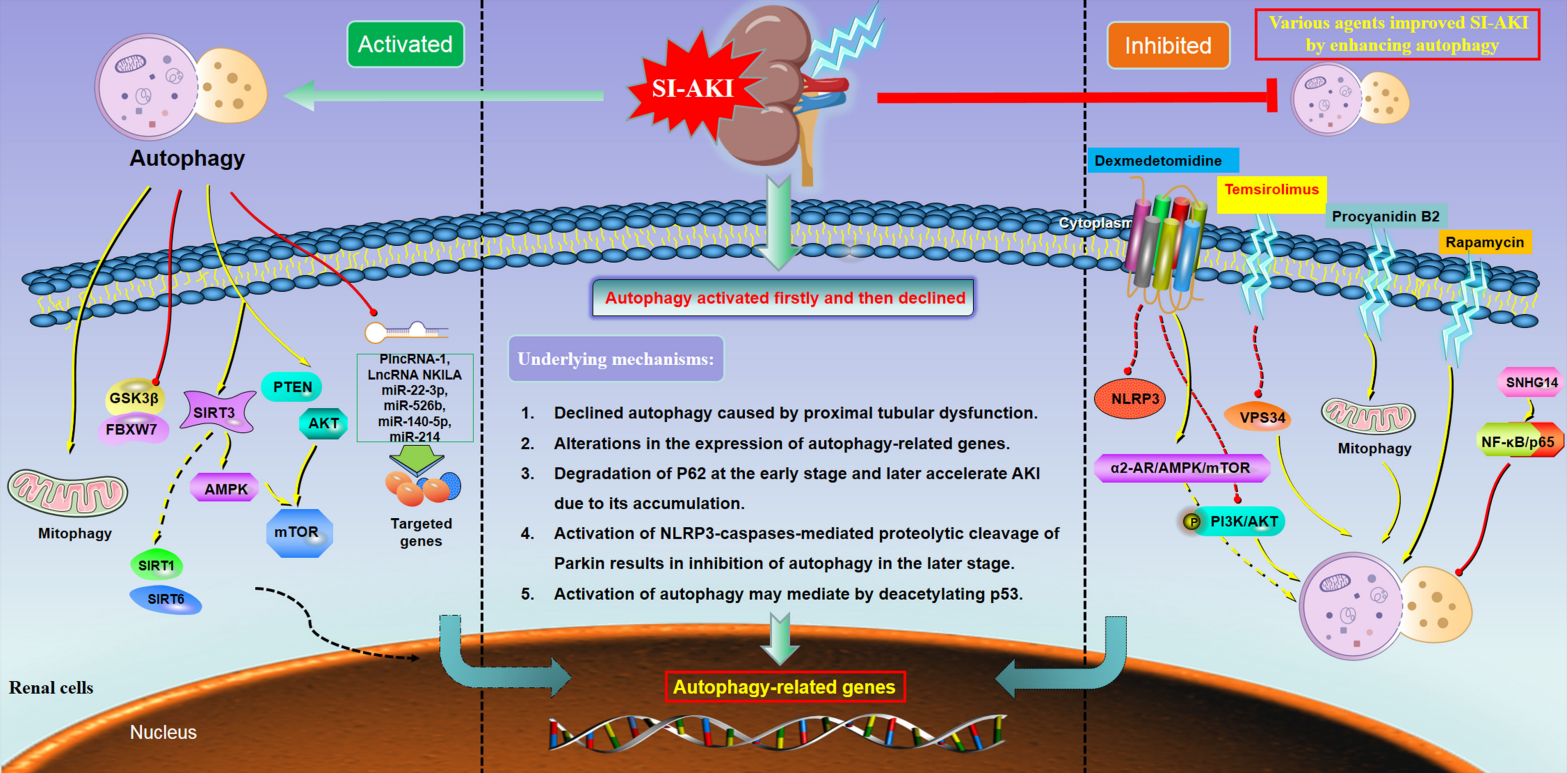
Main molecular mechanisms underlying the dual role of autophagy in sepsis-induced acute kidney injury. Under the condition of SI-AKI, the autophagy level presented with three tendencies, including activation (left column), activated first and then declined (middle column), and inhibition (right column). These distinct patterns of autophagy in SI-AKI might be regulated by multiple associated genes and a series of downstream signaling. Abbreviation: mTOR= mechanistic target of rapamycin; NLRP3, NLR family pyrin domain containing 3; Sirt1, Sirtuin 1; Sirt3, Sirtuin 3; Sirt6, Sirtuin 6; GSK3β, Glycogen Synthase Kinase 3β; AMPK, Adenosine 5’-monophosphate-activated protein kinase; PI3K, Phosphatidylinositol 3-kinase; VPS34, Vacuolar protein sorting 34; PTEN, Phosphatase and tensin homolog.

The characteristics and the main findings of the 24 included studies were summarized in **Table 1**. **Figure 1** (left column) displayed the molecular mechanisms of activated autophagy during SI-AKI.

**Table 1:** The characteristics and the main findings of the 24 relevant studies reporting activation of autophagy in SI-AKI.

Study/Reference	Experimental model/ participant	Methods for establishing sepsis-induced AKI	Status of autophagy	Associated genes or pathways	Main findings
Zhang et al. (14)	Mice	LPS	Augment	Down-regulating GSK3β and FBXW7; Maintaining mTOR expression	CaMKIV signaling mediated the autophagic response to sepsis-induced AKI, by inhibiting GSK3β and FBXW7 expression and maintaining mTOR.
Chen et al. (15)	Mice and HK-2 cells	CLP, LPS	Activated 1 day after CLP	Increasing LC3-II and Rap expression, inhibiting 3-MA protein	Autophagy activation was observed after CLP, while the protective effect developed by Klotho in sepsis-induced AKI might be irrelevant to autophagy.
Fu et al. (16)	NRK-52E cells	LPS	Activated	Up-regulating LC3-II and down-regulating PlncRNA-1 and BCL2	Overexpression of PlncRNA-1 inhibited autophagy by up-regulating BCL2 expression in septic AKI.
Zhao, et al. (18)	Mice	CLP	Activated	Increased LC3-II and BECN1, decreased expression levels of SIRT3	High level of SIRT3 protects against sepsis-induced AKI by modulating AMPK/mTOR-mediated autophagy.
Jia et al. (19)	Rat	CLP	Activated	The expression of LC3II, Atg5, and beclin 1 were significantly increased	Alpha-Lipoic Acid improved the renal functioning in septic AKI by upregulating Atg5, Atg7, and beclin-1 expression, but decreased p62 levels in the kidney.
Wu et al. (20)	Mice	CLP	Activated	Beclin1 and LC3−II/I were significantly elevated	The activation of autophagy might aggravate the renal injury in mice. It was speculated that inhibiting autophagy might increase the survival rate of patients with septic AKI.
Zhang et al. (21)	Mice and HK-2 cells	LPS	Activated	SIRT6 and LC3B-II/LC3B-I expression were significantly increased	Activation of autophagy and increased inflammation were observed in LPS-induced septic AKI. Overexpression of SIRT6 induced autophagy of HK-2 cells.
Zheng et al. (22)	HK-2 cells	LPS	Activated	Increased protein expression levels of beclin-1	Downregulation of ATM significantly inhibited autophagy and inflammatory response in LPS-induced AKI.
Feng et al. (23)	Patients and HK-2 cells	LPS	Activated	Increased level of Beclin-1, LC3-II/I, and NEAT1	Upregulated NEAT1 but downregulated miR-22-3p was observed in patients with sepsis and in LPS-induced HK-2 cells.
Gao et al. (24)	Mice	CLP	Activated	Increased the level of LC3-II but decreased the expression of p62	Polydatin protected against mitochondrial dysfunction in sepsis-induced-AKI by activating mitophagy *via* upregulating SIRT1.
Liu et al. (25)	Mice and HK-2 cells	CLP and LPS	Activated	Increased expression of Beclin-1, LC3-I, LC3-II, and ATG7	ATG7 promoted autophagy in sepsis−induced AKI and was inhibited by miR−526b
Miao et al. (26)	Mice	LPS	Activated	Enhanced LC3-II and 15-PGDH protein expression	Blockade of 15-PGDH promoted autophagic response, alleviating LPS-induced septic AKI.
Alexander et al. (27)	Patients (n=17)	Gene expression analysis	Enhanced	NA	The autophagy level was significantly higher in sepsis AKI than nonsepsis-related AKI (P=0.023), while it was comparable between sepsis AKI and COVID AKI (P=0.621).
Chen et al. (28)	Mice and HK-2 cells	LPS	Activated	Activation of TLR4	Ascorbate protected against LPS-induced AKI by enhancing mitophagy mediated by PINK1-PARK2 axis.
Guo et al. (29)	Rat, HK-2, RTECs cells	CLP and LPS	Activated	Increased LC3II, and decreased level of p62	BMSCs protected rats against sepsis-induced AKI by promoting mitophagy *via* upregulating SIRT1/Parkin.
Han et al. (30)	HK-2 cells	LPS	Activated	Increased LC3II and Beclin1	LncRNA NKILA silencing protected HK-2 cells from sepsis-mediated AKI by decreasing CLDN2 through sponging miR-140-5p.
Li et al. (7)	Mice	LPS	Activated	Increased LC3II and RIP3	RIP3 suppressed autophagic degradation *via* impeding the transcription factor EB -lysosome pathway and the nuclear translocation in septic AKI.
Pan et al. (31)	HK-2 cells	LPS	Activated	Beclin1, ATG5, and LC3B-II was increased; p62 expression was downregulated	Inhibition of TREM-1 increased autophagy in LPS-induced cell model by activating the NF-κB pathway (P-p65, p65, P-IκBα, and IκBα).
Sang et al. (32)	Mice	CLP	Activated	Elevated LC3 II/I and the reduction of p62	Mir-214 protected against sepsis-induced AKI by decreasing oxidative stress and suppressing autophagy *via* regulation of the PTEN/AKT/mTOR pathway.
Tan et al. (33)	Mice	CLP	Activated	The increase of the ratio of LC3−II/I and decrease of the expression of p62	The protective effect of inhibition of aerobic glycolysis against sepsis−induced AKI might be associated with the induction of autophagy *via* the lactate/SIRT3/AMPK pathway.
Wang et al. (34)	Mice	CLP and LPS	Activated	The levels of LC3-II were increased and peaked at 24 h; TOM20 and TIM23 were reduced	Mitophagy was activated in renal tubular cells during septic AKI by up-regulating the autophagy adaptor optineurin (OPTN) expression, which was mediated by the PINK1-PARK2 pathway.
Li et al. (35)	Mice and HK-2 cells	LPS	Activated	The ratio of LC3B−II/LC3B−I increased and the level of p62 decreased	Sodium hydrosulfide hydrate (NaHS) prevented sepsis-associated AKI by promoting autophagy to suppress renal tubular epithelial cell apoptosis and reduce inflammatory factors.
Li et al. (36)	Mice	LPS	Activated	Increased LC3BII expression in platelets	LPS induced platelet autophagy by generating mitochondrial ROS. TLR4 inhibitor TAK242 might effectively alleviate septic AKI by inhibiting platelet GPIIb/IIIa, and reducing platelet activation.
Li et al. (37)	Rat	CLP	Activated	Increased the expression of LC3II	Ulinastatin protected the adhesion junction and ameliorated the perfusion of kidney capillaries during sepsis by the inhibition of autophagy and the up-regulation of VE-cadherin expression.

Atg7, autophagy-related gene 7; LC3, microtubule-associated protein 1 light chain 3; CLP, Cecal ligation and puncture; LPS, Lipopolysaccharide; mTOR, mechanistic target of rapamycin; Pink1, PTEN induced putative kinase 1; Parkin, Parkin RBR E3 ubiquitin protein ligase; NLRP3, NLR family pyrin domain containing 3; HK-2, Human kidney proximal tubular epithelial (HK-2) cells; BMSCs, Bone marrow-derived mesenchymal stem cells; RTECs, Renal tubular epithelial cells; RIP3, Receptor interacting protein kinase 3; ATM, ataxia-telangiectasia mutated; 15-PGDH, 15-hydroxyprostaglandin dehydrogenase; TREM-1, Triggering receptor expressed by myeloid cells; TLR4, Toll Like Receptor 4.

### mTOR signaling pathway

The mammalian target of the rapamycin (mTOR) pathway is one of the pivotal signaling pathways with critical biological function in multiple diseases (59), including SI-AKI (60). mTOR has been reported to play a central role in the regulation of autophagy, characterized by inhibiting autophagy in the biological process of growth factors and abundant nutrients (61). The calcium/calmodulin-dependent protein kinases (CaMK) were found to regulate septic inflammation. Zhang et al. (14) demonstrated that CaMKIV signaling mediated the autophagic response to LPS-associated septic AKI by inhibiting GSK3β and FBXW7 expression and maintaining mTOR levels. mTOR and AMP-activated protein kinase (AMPK) have been reported to correlate with in the development of autophagy in AKI (62). BECN1, Bcl-2, and LC3-II are pivotal autophagy-related proteins. Increased LC3-II and BECN1 expression have been found in SI-AKI, indicating the autophagy status enhanced during sepsis. Zhao et al. (18) showed that elevated autophagy occurred in SI-AKI, while a high level of SIRT3 could protect against AKI by modulating AMPK/mTOR-mediated autophagy. In a mouse model of SI-AKI conducted by CLP, Sang et al. (32) also confirmed that the kidney autophagy level was elevated. They further found that PTEN/AKT/mTOR signaling pathway was involved in this action. The above studies indicated that mTOR was one of the key targets for the activation of autophagy during SI-AKI.

### non-coding RNA

Both long non-coding RNAs (lncRNAs) and microRNA (miRNA) have been suggested to play essential roles in various diseases, including SI-AKI (63, 64). It is known that lncRNA regulates the activities of miRNA through the lncRNA-miRNA interactions. On the topic of the present study, we also found that both lncRNA and miRNA participated in the development of autophagy-mediated SI-AKI. PlncRNA-1 was reported to initiate malignancy in multiple cancers and play roles in inflammatory diseases (65). Fu et al. (16) showed that renal autophagy was activated in SI-AKI by up-regulating LC3-II and down-regulating PlncRNA-1 and BCL2 levels, while overexpression of PlncRNA-1 inhibited autophagy by up-regulating BCL2 expression. Enhanced autophagy was also observed in Feng et al.’ study (23). Upregulated NEAT1 but downregulated miR-22-3p was found in patients with SI-AKI and in LPS-induced HK-2 cells. Liu et al. (23) suggested that ATG7 promoted autophagy (characterized by increasing levels of Beclin-1, LC3-I, and LC3-II) in SI-AKI, which could be inhibited by miR−526b. In a recent study conducted by Han et al. (30), the authors demonstrated that kidney autophagy was activated, while LncRNA NKILA silencing could protect HK-2 cells from SI-AKI by decreasing CLDN2 by sponging miR-140-5p. Sang et al. (32) reported that an increased level of LC3-II was found in CLP-induced SI-AKI, indicating the autophagic level was active. They also observed that miR-214 protected against sepsis-induced AKI by decreasing oxidative stress and suppressing autophagy by regulating the PTEN/AKT/mTOR pathway. These data collectively implied that the enhanced kidney autophagy occurred in SI-AKI, which was partially mediated by several specific non-coding RNAs, e.g. PlncRNA-1, miR-22-3p, miR−526b, LncRNA NKILA, miR-140-5p, and miR-214.

### Mitophagy

Mitophagy, an evolutionarily conserved biological process, is one of the pivotal cytoprotective mechanisms. It functions to remove the damaged mitochondria and maintain a healthy mitochondrial population (66). Gao et al. (24) reported that polydatin could protect against mitochondrial dysfunction in SI-AKI by activating Parkin-dependent mitophagy *via* upregulating SIRT1 and inhibiting NLRP3 activation. Ascorbate is a precursor for carnitine and catecholamine synthesis. It protects against oxidative stress in various diseases (67). Recently, Chen et al. (28) showed that ascorbate protected against LPS-induced AKI by enhancing mitophagy mediated by the PINK1-PARK2 axis. Bone marrow-derived mesenchymal stem cells (BMSCs) play roles in self-renewal and multi-differentiation, functioning in tissue repair and regeneration (68). It was reported that BMSCs protected rats against SI-AKI by promoting mitophagy *via* upregulating SIRT1/Parkin (29). In line with the above studies, Wang et al. (34) also confirmed that autophagy was increased (characterized by a high level of LC3-II) during SI-AKI. They found that mitophagy was activated in renal tubular cells in SI-AKI by up-regulating the autophagy adaptor optineurin (OPTN) expression, which was affected by the PINK1-PARK2 pathway. The above studies demonstrated that several substances (i.e., polydatin, ascorbate, and BMSCs) significantly protected against SI-AKI, which might partially mediate by the activation of mitophagy.

### Sirtuins

Sirtuins belong to the family of NAD^+^-dependent histone deacetylases, which play roles in energy metabolism, inflammation, and tumorigenesis (69). According to some included studies, Sirtuins (e.g. SIRT3, SIRT6, and SIRT1) have been implicated in involving in the activation of autophagy during SI-AKI. Upregulation of SIRT3 has been reported to protect against sepsis-induced AKI (18). Similarly, the protective effects of inhibition of aerobic glycolysis against SI-AKI were found to be associated with the induction of autophagy, which might be partly due to the upregulation of the SIRT3/AMPK pathway (33). In Zhang et al.’s study (21), activation of autophagy (increased LC3B-II/LC3B-I expression) and increased inflammation were observed in LPS-induced septic AKI. The authors further indicated that overexpression of SIRT6 might induce autophagy of HK-2 cells (21). Gao et al. (24) showed that polydatin could protect against mitochondrial dysfunction in SI-AKI by upregulating the expression of SIRT1. These studies suggest that Sirtuins are important in autophagic changes during SI-AKI and are the potential therapeutic targets of SI-AKI.

### Other potential mechanisms

In addition to the above molecular molecules and pathways, the roles of autophagic activation in SI-AKI might also cause or mediate by some other biological agents. Chen et al. (15) showed that SI-AKI was accompanied by increasing LC3-II and Rap expression but inhibiting 3-MA protein. They observed that autophagy activation presented after CLP, while the protective effect developed by Klotho in SI-AKI might be irrelevant to autophagy. Jia et al. (19) reported that Alpha-Lipoic Acid could improve renal functioning in SI-AKI by upregulating the expression of autophagy-associated genes, such as Atg5, Atg7, and beclin-1. It was suggested that Beclin1 and LC3−II/I were significantly increased in SI-AKI, while the activation of autophagy might aggravate the renal injury (20). Zheng et al. (22) also confirmed that autophagy was enhanced in SI-AKI. They next found that downregulation of ataxia-telangiectasia mutated (ATM) significantly suppressed autophagy and inflammatory response in LPS-induced AKI. 15-hydroxyprostaglandin dehydrogenase (15-PGDH) is an important enzyme in the degradation of prostaglandins. Miao et al. (26) demonstrated that the blockade of 15-PGDH promoted autophagic response, alleviating LPS-induced SI-AKI. Receptor interacting-protein kinase 3 (RIP3) has been found to function as protect against renal tubular injury and renal dysfunction during septic AKI. Li et al. (7) revealed that RIP3 suppressed autophagic degradation by impeding the transcription factor EB-lysosome pathway and the nuclear translocation in SI-AKI.

Triggering receptor expressed by myeloid cells (TREM-1) is an amplifier of inflammatory responses induced by infections. Pan et al. demonstrated that inhibition of TREM-1 elevated autophagy in SI-AKI by activating the NF-κB pathway (P-p65, p65, P-IκBα, and IκBα). Sodium hydrosulfide hydrate (NaHS) has been implicated in preventing SI-AKI by promoting autophagy to suppress renal tubular epithelial cell apoptosis and reduce inflammatory factors (35). LPS can increase the production of intracellular ROS *via* Toll-like Receptor 4 (TLR4), which can lead to mitochondrial damage and activate platelets. LPS-induced platelet autophagy by generating mitochondrial ROS. Li et al. (36) showed that TLR4 inhibitor TAK242 might effectively alleviate SI-AKI by inhibiting platelet GPIIb/IIIa and platelet activation. Ulinastatin, a urinary trypsin inhibitor, functions to control a series of proinflammatory mediators and cytokines. A recent study showed that Ulinastatin protected the adhesion junction and ameliorated the perfusion of kidney capillaries during SI-AKI by suppressing autophagy and elevating VE-cadherin expression (37). Taken together, all the above-mentioned genes and substances might be involved in the biogenesis and biological functions of autophagy activation in SI-AKI. Targeting these affected proteins may be one of the effective therapeutic regimens that can protect against SI-AKI.

In summary, the above 24 included studies demonstrated that autophagy was increased during SI-AKI, while some drugs, substances, or molecules exert protective effects against SI-AKI also due to their properties on the promotion and enhancement of autophagy. One possible mechanism is that increased autophagy may be one of the phenotypes of the early stage of SI-AKI, which can be considered as a physiological compensatory response. Nevertheless, some specific external interventions applied for enhancing the autophagy flux may contribute to the renoprotective effects on SI-AKI, which may be partially related to a high level of autophagy in the later stage of SI-AKI may promote renal cell survival.

#### Autophagic inhibition in SI-AKI

Among the 41 included studies, a majority of them indicated that the autophagy flux was increased in SI-AKI, while there were eight studies (8/41, 19.5%) suggested that autophagy was inhibited during SI-AKI (**Table 2**). These experimental animal and cellular models indicated that autophagy was diminished in the kidney of SI-AKI and that proximal tubule cells fail to promote autophagy. Since the inhibition of autophagy was observed in SI-AKI, enhanced autophagy flux may effectively improve renal recovery.

**Table 2:** The characteristics and the main findings of the 9 relevant studies reporting inhibition of autophagy in SI-AKI.

Study/Reference	Experimental model/ participant	Methods for establishing sepsis-induced AKI	Status of autophagy	Associated genes or pathways	Main findings
Howell et al. (38)	Mice	CLP; LPS administration	Inhibited	Inhibition of LC3b and VPS34 expression and activation of mTOR	During sepsis, diminished autophagy was associated with renal dysfunction, while treatment with temsirolimus or inhibiting VPS34 expression significantly improve renal function by elevating autophagy.
Sunahara, et al. (48)	Mice	CLP	Inhibited	The number of autophagosomes decreased at 24h after CLP	Autophagy significantly reduced in the kidney during the acute phase of sepsis. Rapamycin could improve the renal functioning by accelerating autophagy.
Li et al. (40)	HK-2 and HEK-293 cells	LPS	Inhibited	Decreasing the levels of LC3II, but increasing the p62 expression through the AMPK/SIRT1 pathway	Recombinant human erythropoietin (rhEPO) alleviated septic AKI by activating autophagy through AMPK/SIRT1 pathway.
Feng et al. (23)	Rat	LPS	Inhibited	Decreased expression of mfn2, PINK1, Parkin, and LC3-II, while increased the level of Drp1 and p62, which mediated by inhibiting Nrf2	Treatment with human umbilical cord blood mononuclear cells (hUCBMNCs) protected against LPS-induced AKI by increasing autophagy in kidney.
Liu et al. (25)	Mice	LPS	Mitophagy was decreased	The levels of LC3, Pink1, and Parkin were decreased, while the expression of TOM20 and TIM23 were increased	The protective effects of antioxidant Procyanidin B2 on mitochondrial dynamics in septic AKI might partially through the elevation of mitophagy level, which might be associated with the increased nuclear translocation of Nrf2.
Yang et al. (43)	Rat	LPS	Inhibited	Decreased expression of LC3-II, beclin-1, and NLRP3, but increased expression of p62	Dexmedetomidine protected against LPS-induced AKI by enhancing autophagy *via* inhibiting NLRP3 inflammasome and activating α2-AR/AMPK/mTOR pathway.
Zhao et al. (44)	Rat	LPS	Inhibited	Decreased the expression of Beclin-1, LC3 II, and PINK1	Dexmedetomidine ameliorated LPS-induced AKI by enhancing autophagy through inhibiting the phosphorylation levels of PI3K, AKT, and mTOR.
Yang et al. (53)	HK-2 cells	LPS	Inhibited	Decreased LC3-II/LC3-I ratio and enhanced p62 expression	SNHG14 inhibited cell autophagy and promoted inflammatory cytokine production in a SI-AKI cell model. SNHG14/miR-495-3p/HIPK1 interaction network played role in this action *via* modulating NF-κB/p65 signaling.
Yu et al. (45)	Mice	LPS	Inhibited	Elevated the protein expression of LC3A and p62	NF-κB inhibitor 270 protected against septic AKI by promoting autophagy *via* the inhibition of NF-κB transcriptional activity, NF-κB, and JNK signaling pathways mediated inflammation responses.

LC3, microtubule-associated protein 1 light chain 3; CLP, Cecal ligation and puncture; LPS, Lipopolysaccharide; mTOR, mechanistic target of rapamycin; Pink1, PTEN induced putative kinase 1; Parkin, Parkin RBR E3 ubiquitin protein ligase; NLRP3, NLR family pyrin domain containing 3; HK-2, Human kidney proximal tubular epithelial (HK-2) cells; AR, Androgen receptor.

Howell et al. (38) found that diminished autophagy was associated with renal dysfunction during SI-AKI. Meanwhile, the authors also observed that VPS34 expression was inhibited and the mTOR was activated. Further, they discovered that SI-AKI animals treated with temsirolimus (an mTOR inhibitor) or inhibiting VPS34 expression significantly improve renal function by elevating autophagy. Yang et al. (53) reported that the autophagy level was inhibited during SI-AKI, which was characterized by decreased LC3-II/LC3-I ratio and enhanced p62 expression. In this study, SNHG14 inhibited cell autophagy and promoted inflammatory cytokine production in SI-AKI. SNHG14/miR-495-3p/HIPK1 interaction network plays a key role in the septic process, which might be modulated *via* the NF-κB/p65 signaling. Rapamycin, an inducer of autophagy, has been found to reduce the extent of SI-AKI. Sunahara et al. (48) reported that the number of autophagosomes decreased at 24h after CLP, indicating that autophagy was restrained during SI-AKI. They next found that rapamycin could improve renal functioning by accelerating autophagy. Li et al. (40) showed that the levels of LC3II were reduced in SI-AKI compared to the controls, but the expression of p62 was increased. The authors revealed that recombinant human erythropoietin (rhEPO) could alleviate SI-AKI by activating autophagy through AMPK/SIRT1 pathway. Feng et al. (50) reported that treatment with human umbilical cord blood mononuclear cells (hUCBMNCs) protected against LPS-induced AKI by increasing autophagy in the kidney. The underlying mechanisms might be associated with the decreased expression of several proteins (e.g. mfn2, PINK1, Parkin, and LC3-II) and the elevated expression of Drp1 and p62, which might be partly mediated by inhibiting Nrf2. A more recent study developed by Yu et al. (45) showed that NF-κB inhibitor 270 could protect against SI-AKI by promoting autophagy by reducing inflammation responses, which might be associated with the inhibition of NF-κB transcriptional activity, NF-κB, and JNK signaling pathways.

Mitophagy also plays a key role in the inhibition of autophagy in SI-AKI. Procyanidin B2 (PB2), one of the common antioxidants, exerts excellent anti-oxidative and anti-inflammatory effects on multiple diseases (70). Decreased LC3, Pink1, and Parkin, while increased TOM20 and TIM23 were identified in Liu et al.’s study (42). The authors further suggested that the protective effects of antioxidant Procyanidin B2 on mitochondrial dynamics in SI-AKI might be partially through the elevation of mitophagy level, which might be associated with the increased nuclear translocation of Nrf2.

Dexmedetomidine (DEX), a selective α2-adrenoreceptor agonist, functions with the effects of the sedative, analgesic, and anti-anxiety (71). Besides, mounting experimental studies demonstrated that DEX has outstanding antioxidant, anti-apoptosis, and anti-inflammatory effects (72). Yang et al. (43) reported that the autophagy level was decreased in SI-AKI (characterized by decreased expression of LC3-II and Beclin-1). The investigators subsequently found that DEX protected against LPS-induced AKI by enhancing autophagy, which might be correlated to the inhibition of NLRP3 inflammasome and the activation of the α2-AR/AMPK/mTOR pathway. Consistent with Yang et al.’ study, Zhao also observed a reduced autophagy flux during SI-AKI (characterized by decreased Beclin-1 and LC3 II expression). They showed that DEX ameliorated LPS-induced AKI by promoting autophagy by inhibiting the phosphorylation levels of PI3K, AKT, and mTOR.

Taken together, the above eight included studies demonstrated that the autophagy level was decreased during SI-AKI, while the promotion of autophagy flux might significantly improve the renal function, which suggested that autophagy played a protective role against SI-AKI. The mechanisms of autophagy inhibition in SI-AKI and the potential molecular mechanisms were illustrated in **Figure 1** (right column).

### Autophagy rises first and then falls during SI-AKI

Within the topic of this study, nine included studies demonstrated that autophagy rose firstly and decreased later in SI-AKI (**Table 3**). Besides, some eligible studies also showed that the autophagy level increased early, then declined, and increased again later. Autophagy is commonly upregulated by environmental stress, such as inflammatory mediators, mitochondrial dysfunction, and ATP depletion, to maintain homeostasis (73). In addition, the autophagy process is a tightly regulated machinery, which can remove damaged proteins and organelles (74). According to the available data from the included studies in this study, the early stage of SI-AKI could be defined as less than 8h after CLP or LPS treatment, while sepsis >8h could be thought as the late stage of SI-AKI. Hsiao et al. (46) observed that the expression of LC3-II increased at 3h and 6 h after CLP but sequentially decreased to the basal level at 9h and 18 h after CLP. In response to septic insult, the level of autophagy transiently elevated in kidney tissue at CLP_3h_. Due to renal dysfunction and morphological injury, renal autophagy declined at late sepsis, which contributed to proximal tubular dysfunction in an animal model of SI-AKI. *In vitro* study, siRNA knockdown of Atg7 on NRK-52E cells significantly declined the level of LC3-II. This is the first *in vivo* study to detect the decline of autophagy that may be conducive to the pathogenesis of polymicrobial sepsis-mediated AKI. Since then, several following preclinical studies also demonstrated a trend of rising first and then falling of autophagy during SI-AKI.

**Table 3:** The characteristics and the main findings of the relevant 8 studies reporting autophagy is activated firstly and then declined in SI-AKI.

Study/Reference	Experimental model/ participant	Methods for establishing sepsis-induced AKI	Status of autophagy	Associated genes or pathways	Main findings
Hsiao et al. (46)	Rat and NRK-52E	CLP	Increased autophagy in early sepsis and inhibited at 9h and 18h after CLP	Up-regulating LC3-II and Atg7	Increased LC3-II at 3h and 6 h after CLP and sequentially decreased to the basal level at 9h and 18 h after CLP. *In vitro* study, siRNA knockdown of Atg7 on NRK-52E cells significantly declined the level of LC3-II. Declination of autophagy contributed to proximal tubular dysfunction at the late stage of sepsis.
Karagiannidis et al. (39)	Rat	CLP	Autophagy increased at 6 h after sepsis and declined at 12 and 24h, while elevated at 36 h	LC3a/b and pERK expression enhanced at the early sepsis, then declined, and increased later, while pAKT expression had a contrary tendency.	Autophagy inductions might be a cytoprotective mechanism triggered under sepsis conditions, rather than an alternative cell death pathway. These results provided a new prospective in sepsis treatment.
Mei et al. (47)	Mice	LPS	Autophagy increased at 4-24h after sepsis and declined to the control level subsequently.	LC3 II was increased at 4-24h after LPS treatment, then reduced to the normal level.	LPS-induced renal autophagy is suppressed in Atg7- knockout animal. Besides, more severe AKI was observed in proximal tubule-specific Atg7-knockout mice.
Li et al. (17)	Mice	LPS	Activated firstly and then declined	Increased expression levels of LC3-II, reduced P62 expression at early stage	The degradation of P62 by activated autophagy at the early stages of endotoxemia resulted in the inhibition of apoptosis; At the late stages of endotoxemia, inhibition of autophagy caused P62 accumulation and accelerated renal injury.
Dai et al. (49)	HK-2 cells and Rat	LPS	First activated and then inhibited	Activated LC3-II, BECN-1, and PINK1-Parkin pathway	Mitophagy increased with the first 4h after LPS stimulation and was decreased thereafter. Mitophagy protected LPS-included cells from apoptosis, and improved renal functions of rats with septic AKI.
Liu et al. (25)	Mice	CLP	Elevated in early stage but declined in the later phase	Elevated levels of LC3, COX IV, Pink1, Parkin, and NLRP3	Impaired mitophagy in the later stage of septic AKI might be correlated with the activation of NLRP3-caspases-mediated proteolytic cleavage of Parkin.
Deng et al. (51)	Mice	CLP	First activated and then declined	Inhibited Beclin-1 expression	SIRT1 activation improved sepsis AKI by promoting Beclin1-mediated autophagy.
Sun et al. (52)	Mice	CLP	Activated and then returned to normal level	LC3II elevated gradually and peaked at 8 h and returned to baseline by 24 h	Sirt1 upregulation reduced sepsis-induced AKI by deacetylating p53 to activate autophagy.

AKI, Acute kidney injury; Atg7, autophagy-related gene 7; LC3, microtubule-associated protein 1 light chain 3; CLP, Cecal ligation and puncture; LPS, Lipopolysaccharide; mTOR, mechanistic target of rapamycin; Pink1, PTEN induced putative kinase 1; Parkin, Parkin RBR E3 ubiquitin protein ligase; NLRP3, NLR family pyrin domain containing 3; HK-2, Human kidney proximal tubular epithelial (HK-2) cells; Sirt1, Sirtuin 1; COX IV, cytochrome coxidase IV.

Mei et al. (47) showed that autophagy increased at 4-24h after sepsis and declined to the control level subsequently. LPS-induced renal autophagy was suppressed in Atg7-knockout animals. Additionally, more severe AKI was observed in proximal tubule-specific Atg7-knockout mice. Since the aberrant expression of autophagy-related genes significantly affects the autophagy status under sepsis, this fact may imply that autophagy plays an essential role in SI-AKI. Increased expression levels of LC3-II and reduced P62 expression at an early stage were observed in Li et al.’s study (17). The authors further pointed out that the degradation of P62 by activated autophagy at the early stages of endotoxemia might induce the inhibition of apoptosis. At the late stages of endotoxemia, inhibition of autophagy caused P62 accumulation and accelerated renal injury. Similarly, Dai et al. (49) also found that the autophagy level was first activated and then inhibited during SI-AKI. Mitophagy was increased within the first 4h after LPS stimulation and was decreased thereafter. Mitophagy protected LPS-included cells from apoptosis and improved renal functions of SI-AKI.

Under a similar trend of the autophagy change (increased first and then decreased) in SI-AKI, elevated levels of LC3, COX IV, Pink1, Parkin, and NLRP3 were identified in septic AKI (41). Impaired mitophagy in the later stage of septic AKI might be correlated with the activation of NLRP3-caspases-mediated proteolytic cleavage of Parkin. Sirtuin 1 (Sirt1), an NAD^+^-dependent protein deacetylase, functions to modify deacetylate histone and nonhistone proteins. Consistently, Deng et al. (51) also demonstrated that kidney autophagy was elevated in the early stage but declined in the later phase. The researchers also found that SIRT1 activation improved SI-AKI by promoting Beclin1-mediated autophagy. A recent study conducted by Sun et al. (52) indicated that the expression of LC3II elevated gradually and peaked at 8 h and returned to baseline by 24 h after SI-AKI, indicating the autophagy level increased first and then declined to normal level subsequently. The authors then showed that Sirt1 upregulation reduced SI-AKI by deacetylating p53 to activate autophagy. Differ from that of the above included studies reporting the autophagy level increased at the early stage and decreased later during SI-AKI, Karagiannidis et al. implied that the autophagy flux increased at 6 h after sepsis and declined at 12 and 24h, while elevated at 36 h. They concluded that autophagy inductions might be a cytoprotective mechanism triggered under sepsis conditions, rather than an alternative cell death pathway.

Based on the above evidence derived from *in vitro* and *in vivo* models of SI-AKI, it showed the trend of rising firstly and then falling for the autophagy flux. This may be a molecular mechanism of the renoprotective effect during SI-AKI (**Figure 1**, middle column). The autophagy feedback may play a protective role in endotoxic AKI, serving as a potential therapeutic target for protecting against the damage of renal tubular epithelial cells.

### Hypotheses for the different level of autophagy among the included studies

Based on the different level of autophagy among the 41 included studies and outcomes of the changed autophagic flux, we propose the following hypotheses. First, the autophagic flux during the physiological processes of SI-AKI might elevate firstly (phrase I), next inhibit or decline (phrase II), then elevate (phrase III), and return to the normal level finally (phrase IV). In the 24 studies reporting the elevation of autophagy, it could be explained by the check point time of autophagy in these studies was phrase I or phrase III. The eight studies reporting the elevation first then inhibition could be explained by check point time of autophagy was phrase I and phrase II of SI-AKI. Of note, in the nine included studies reported elevated first and then declined, all of them concluded that the autophagy status was inhibited during SI-AKI, while the renal protective effects exerting by specific interventions (reported in eight studies) might be contributed to the elevation of autophagy. Therefore, the check point time of autophagy in these included studies might be the phrase II. The above interventions exerted the renoprotective effects on SI-AKI might be associated with the acceleration of autophagy from phrase II to phrase III.

### The changes of autophagic flux under different interventions in the experimental models of SI-AKI

As shown in the **Table 4**, the majority of the included studies (14/15, 93%) showed that the improvement of SI-AKI exhibited by specific interventions might be attributed to the elevation of autophagy, regardless of whether the autophagy activated or inhibited during SI-AKI. The most likely explanation is the elevation of autophagy may be one of the protective mechanisms for SI-AKI. Another possible explanation is that the activation and inhibition of autophagy among different studies might be associated with the different time points of examinations after sepsis in each independent study. As illustrated in **Table 3**, eight included studies indicated the autophagy was activated firstly (early stage of SI-AKI) and then declined (late stage of SI-AKI) during SI-AKI. Therefore, those studies listed in **Table 1** suggested that autophagy was activated, which might be due to the time point for checking the autophagy level being the early stage. On the other hand, the inhibition of autophagy in the studies listed in **Table 2** might be correlated to the checking time point was the late stage of SI-AKI.

**Table 4:** The status of autophagy under different interventions in the experimental models of SI-AKI.

Study/Reference	Experimental model/ participant	Methods for establishing sepsis-induced AKI	Specific interventions	Status of autophagy without interventions	Status of autophagy under interventions
Jia et al. (19),	Rat	CLP	Alpha-Lipoic Acid	Activated	Activated
Gao et al. (24),	Mice	CLP	Polydatin	Activated	Activated
Chen et al. (28),	Mice and HK-2 cells	LPS	Ascorbate	Activated	Activated
Guo et al. (29)	Rat, HK-2, RTECs cells	CLP and LPS	BMSCs	Activated	Activated
Tan et al. (33)	Mice	CLP	Aerobic glycolysis	Activated	Activated
Li et al. (35)	Mice and HK-2 cells	LPS	Sodium hydrosulfide hydrate	Activated	Activated
Li et al. (37)	Rat	CLP	Ulinastatin	Activated	Inhibited
Howell et al. (38)	Mice	CLP and LPS	temsirolimus	Inhibited	Activated
Sunahara et al. (48)	Mice	CLP	Rapamycin	Inhibited	Activated
Li et al. (40)	HK-2 and HEK-293 cells	LPS	Recombinant human erythropoietin	Inhibited	Activated
Feng et al. (23)	Rat	LPS	umbilical cord blood mononuclear cells	Inhibited	Activated
Liu et al. (25)	Mice	LPS	Procyanidin B2	Inhibited	Activated
Yang et al. (43)	Rat	LPS	Dexmedetomidine	Inhibited	Activated
Zhao et al. (44),	Rat	LPS	Dexmedetomidine	Inhibited	Activated
Yu et al. (45)	Mice	LPS	NF-κB inhibitor 270	Inhibited	Activated

In **Table 1**, there were seven studies reported some interventions for treating SI-AKI. All of the seven included studies indicated CLP or LPS treatment (methods for SI-AKI model establishment) might induce the elevation of autophagy level. Six of them reported that the specific interventions (i.e., alpha-lipoic acid, Polydatin, Ascorbate, BMSCs, aerobic glycolysis, and sodium hydrosulfide hydrate) exhibited the renal protection by increasing autophagy. In **Table 2**, eight studies reported some interventions for treating SI-AKI. Inconsistent with the above findings, all the eight studies concluded that the autophagy status was inhibited during SI-AKI, while the renal protective effects exerting by specific interventions (i.e., temsirolimus, rapamycin, recombinant human erythropoietin, umbilical cord blood mononuclear cells, Procyanidin B2, dexmedetomidine, and NF-κB inhibitor 270) might be contributed by the elevation of autophagy.

Based on the above evidence, in the aspect of the clinical translational perspective, the strategies for therapeutic intervention should focus on how to elevate the autophagic flux during SI-AKI.

## Conclusion and perspectives

To the best of our knowledge, this is the first systematic and comprehensive review to summarize all the current evidence of the crucial roles of autophagy in SI-AKI. We can notice that a majority of the included studies (about 60%) showed an elevation of the autophagy level during SI-AKI, while 22% and 19.5% of the included studies reported an inhibition and an elevation at the early stage but a declination of renal autophagy in SI-AKI, respectively. As can be seen, the level of autophagy flux in the process of septic AKI is still controversial among different studies. One of the explanations for this inconsistency of the autophagy level in SI-AKI may be caused by the various time points monitored in each study. In addition, different intracellular signaling molecules and pathways involved in the process of SI-AKI may also affect the expression of the autophagy-related genes, resulting in an increase or decrease of autophagy flux. Autophagy is considered to be a “double-edged sword” for both cell survival and cell death in multiple diseases, including SI-AKI. However, this study highlights that one of the main probable mechanisms underlying the multiple treatment modalities (e.g. small molecule inhibitors, temsirolimus, rapamycin, ascorbate, rhEPO, stem cells, Procyanidin B2, and DEX) for improving the renal function may be attributed to the elevation of the autophagy level in SI-AKI. The exact roles of autophagy in SI-AKI have not been well understood, which deserves further investigation. Targeting the autophagy-associated proteins and pathways may provide a new prospective in the treatment of critically ill patients with SI-AKI, but more preclinical studies are still warranted to validate this hypothesis.

## Author contributions

SZ, JL, and MW contributed to conceiving and designing the study. XL performed the systematic searching. MLS extracted the data. SZ and JL wrote the manuscript. XL and MW supervised the manuscript. All authors contributed to the article and approved the submitted version.

## Glossary

**Table:**

Atg7	autophagy-related gene 7
LC3	microtubule-associated protein 1 light chain 3
CLP	Cecal ligation and puncture
LPS	Lipopolysaccharide
mTOR	mechanistic target of rapamycin
Pink1	PTEN induced putative kinase 1
Parkin	Parkin RBR E3 ubiquitin protein ligase
NLRP3	NLR family pyrin domain containing 3
HK-2	Human kidney proximal tubular epithelial (HK-2) cells
BMSCs	Bone marrow-derived mesenchymal stem cells
RTECs	Renal tubular epithelial cells
RIP3	Receptor interacting protein kinase 3
ATM	ataxia-telangiectasia mutated
15-PGDH	15-hydroxyprostaglandin dehydrogenase
TREM-1	Triggering receptor expressed by myeloid cells
TLR4	Toll Like Receptor 4
Pink1	PTEN induced putative kinase 1
Parkin	Parkin RBR E3 ubiquitin protein ligase
NLRP3	NLR family pyrin domain containing 3
HK-2	Human kidney proximal tubular epithelial (HK-2) cells
AR	Androgen receptor
COX IV	cytochrome coxidase IV

## References

[1] Liu V Escobar GJ Greene JD Soule J Whippy A Angus DC. Hospital deaths in patients with sepsis from 2 independent cohorts. JAMA (2014) 312:90–2. doi: 10.1001/jama.2014.5804 24838355

[2] Gando S Shiraishi A Yamakawa K Ogura H Saitoh D Fujishima S. Role of disseminated intravascular coagulation in severe sepsis. Thromb Res (2019) 178:182–8. doi: 10.1016/j.thromres.2019.04.025 31054468

[3] He FF Wang YM Chen YY Huang W Li ZQ Zhang C. Sepsis-induced AKI: from pathogenesis to therapeutic approaches. Front Pharmacol (2022) 13:981578. doi: 10.3389/fphar.2022.981578 36188562 PMC9522319

[4] Peerapornratana S Manrique-Caballero CL Gomez H Kellum JA. Acute kidney injury from sepsis: current concepts, epidemiology, pathophysiology, prevention and treatment. Kidney Int (2019) 96:1083–99. doi: 10.1016/j.kint.2019.05.026 PMC6920048 31443997

[5] Huang G Bao J Shao X Zhou W Wu B Ni Z. Inhibiting pannexin-1 alleviates sepsis-induced acute kidney injury via decreasing NLRP3 inflammasome activation and cell apoptosis. Life Sci (2020) 254:117791. doi: 10.1016/j.lfs.2020.117791 32416166

[6] Lorencio CC Yebenes JC Vela E Cleries M Sirvent JM Fuster-Bertolin C. Trends in mortality in septic patients according to the different organ failure during 15 years. Crit Care (2022) 26:302. doi: 10.1186/s13054-022-04176-w 36192781 PMC9528124

[7] Li R Zhao X Zhang S Dong W Zhang L Chen Y. RIP3 impedes transcription factor EB to suppress autophagic degradation in septic acute kidney injury. Cell Death Dis (2021) 12:593. doi: 10.1038/s41419-021-03865-8 34103472 PMC8187512

[8] Adriaenssens E Ferrari L Martens S. Orchestration of selective autophagy by cargo receptors. Curr Biol (2022) 32:R1357–71. doi: 10.1016/j.cub.2022.11.002 36538890

[9] Vargas J Hamasaki M Kawabata T Youle RJ Yoshimori T. The mechanisms and roles of selective autophagy in mammals. Nat Rev Mol Cell Biol (2023) 24:167–185. doi: 10.1038/s41580-022-00542-2 36302887

[10] Levine B Kroemer G. Biological functions of autophagy genes: a disease perspective. CELL (2019) 176:11–42. doi: 10.1016/j.cell.2018.09.048 30633901 PMC6347410

[11] Bhatia D Choi ME. Autophagy in kidney disease: advances and therapeutic potential. Prog Mol Biol Transl Sci (2020) 172:107–33. doi: 10.1016/bs.pmbts.2020.01.008 32620239

[12] Kawakami T Gomez IG Ren S Hudkins K Roach A Alpers CE. Deficient autophagy results in mitochondrial dysfunction and FSGS. J Am Soc NEPHROL. (2015) 26:1040–52. doi: 10.1681/ASN.2013111202 PMC4413752 25406339

[13] Choi ME. Autophagy in kidney disease. Annu Rev Physiol (2020) 82:297–322. doi: 10.1146/annurev-physiol-021119-034658 31640469

[14] Zhang X Howell GM Guo L Collage RD Loughran PA Zuckerbraun BS. CaMKIV-dependent preservation of mTOR expression is required for autophagy during lipopolysaccharide-induced inflammation and acute kidney injury. J Immunol (2014) 193:2405–15. doi: 10.4049/jimmunol.1302798 PMC4215705 25070845

[15] Chen X Tong H Chen Y Chen C Ye J Mo Q. Klotho ameliorates sepsis-induced acute kidney injury but is irrelevant to autophagy. Onco Targets Ther (2018) 11:867–81. doi: 10.2147/OTT.S156891 PMC5823070 29497318

[16] Fu D Zhou K Liu J Zheng P Li P Cheng W. Long non-coding RNA PlncRNA-1 regulates cell proliferation, apoptosis, and autophagy in septic acute kidney injury by regulating BCL2. Int J Clin Exp Pathol (2018) 11:314–23. PMC6957948 31938114

[17] Li T Zhao J Miao S Xu Y Xiao X Liu Y. Dynamic expression and roles of sequestome−1/p62 in LPS−induced acute kidney injury in mice. Mol Med Rep (2018) 17:7618–26. doi: 10.3892/mmr.2018.8809 PMC5983950 29620262

[18] Zhao W Zhang L Chen R Lu H Sui M Zhu Y. SIRT3 protects against acute kidney injury *via* AMPK/mTOR-regulated autophagy. Front Physiol (2018) 9:1526. doi: 10.3389/fphys.2018.01526 30487750 PMC6246697

[19] Jia J Gong X Zhao Y Yang Z Ji K Luan T. Autophagy enhancing contributes to the organ protective effect of alpha-lipoic acid in septic rats. Front Immunol (2019) 10:1491. doi: 10.3389/fimmu.2019.01491 31333648 PMC6615199

[20] Wu Y Wang L Meng L Cao GK Zhao YL Zhang Y. Biological effects of autophagy in mice with sepsis-induced acute kidney injury. Exp Ther Med (2019) 17:316–22. doi: 10.3892/etm.2018.6899 PMC6307358 30651797

[21] Zhang Y Wang L Meng L Cao G Wu Y. Sirtuin 6 overexpression relieves sepsis-induced acute kidney injury by promoting autophagy. Cell Cycle (2019) 18:425–36. doi: 10.1080/15384101.2019.1568746 PMC6422466 30700227

[22] Zheng C Zhou Y Huang Y Chen B Wu M Xie Y. Effect of ATM on inflammatory response and autophagy in renal tubular epithelial cells in LPS-induced septic AKI. Exp Ther Med (2019) 18:4707–17. doi: 10.3892/etm.2019.8115 PMC6862447 31777559

[23] Feng Y Liu J Wu R Yang P Ye Z Song F. NEAT1 aggravates sepsis-induced acute kidney injury by sponging miR-22-3p. Open Med (Wars). (2020) 15:333–42. doi: 10.1515/med-2020-0401 PMC7712373 33335994

[24] Gao Y Dai X Li Y Li G Lin X Ai C. Role of parkin-mediated mitophagy in the protective effect of polydatin in sepsis-induced acute kidney injury. J Transl Med (2020) 18:114. doi: 10.1186/s12967-020-02283-2 32131850 PMC7055075

[25] Liu Y Xiao J Sun J Chen W Wang S Fu R. ATG7 promotes autophagy in sepsis−induced acute kidney injury and is inhibited by miR−526b. Mol Med Rep (2020) 21:2193–201. doi: 10.3892/mmr.2020.11001 PMC7115197 32323768

[26] Miao S Lv C Liu Y Zhao J Li T Wang C. Pharmacologic blockade of 15-PGDH protects against acute renal injury induced by LPS in mice. Front Physiol (2020) 11:138. doi: 10.3389/fphys.2020.00138 32231583 PMC7082810

[27] Alexander MP Mangalaparthi KK Madugundu AK Moyer AM Adam BA Mengel M. Acute kidney injury in severe COVID-19 has similarities to sepsis-associated kidney injury: a multi-omics study. MAYO Clin Proc (2021) 96:2561–75. doi: 10.1016/j.mayocp.2021.07.001 PMC8279954 34425963

[28] Chen ZD Hu BC Shao XP Hong J Zheng Y Zhang R. Ascorbate uptake enables tubular mitophagy to prevent septic AKI by PINK1-PARK2 axis. Biochem Biophys Res Commun (2021) 554:158–65. doi: 10.1016/j.bbrc.2021.03.103 33798942

[29] Guo J Wang R Liu D. Bone marrow-derived mesenchymal stem cells ameliorate sepsis-induced acute kidney injury by promoting mitophagy of renal tubular epithelial cells via the SIRT1/Parkin axis. Front Endocrinol (Lausanne). (2021) 12:639165. doi: 10.3389/fendo.2021.639165 34248837 PMC8267935

[30] Han D Fang R Shi R Jin Y Wang Q. LncRNA NKILA knockdown promotes cell viability and represses cell apoptosis, autophagy and inflammation in lipopolysaccharide-induced sepsis model by regulating miR-140-5p/CLDN2 axis. Biochem Biophys Res Commun (2021) 559:8–14. doi: 10.1016/j.bbrc.2021.04.074 33932903

[31] Pan P Liu X Wu L Li X Wang K Wang X. TREM-1 promoted apoptosis and inhibited autophagy in LPS-treated HK-2 cells through the NF-kappaB pathway. Int J Med Sci (2021) 18:8–17. doi: 10.7150/ijms.50893 33390769 PMC7738954

[32] Sang Z Dong S Zhang P Wei Y. miR−214 ameliorates sepsis−induced acute kidney injury *via* PTEN/AKT/mTOR−regulated autophagy. Mol Med Rep (2021) 24:683. doi: 10.3892/mmr.2021.12322 34328194 PMC8365606

[33] Tan C Gu J Li T Chen H Liu K Liu M. Inhibition of aerobic glycolysis alleviates sepsis−induced acute kidney injury by promoting lactate/Sirtuin 3/AMPK−regulated autophagy. Int J Mol Med (2021) 47:19. doi: 10.3892/ijmm.2021.4852 33448325 PMC7849980

[34] Wang Y Zhu J Liu Z Shu S Fu Y Liu Y. The PINK1/PARK2/optineurin pathway of mitophagy is activated for protection in septic acute kidney injury. Redox Biol (2021) 38:101767. doi: 10.1016/j.redox.2020.101767 33137712 PMC7606859

[35] Li T Zhao J Miao S Chen Y Xu Y Liu Y. Protective effect of H(2)S on LPS−induced AKI by promoting autophagy. Mol Med Rep (2022) 25:96. doi: 10.3892/mmr.2022.12612 35059738 PMC8809055

[36] Li Y Feng G. TLR4 inhibitor alleviates sepsis-induced organ failure by inhibiting platelet mtROS production, autophagy, and GPIIb/IIIa expression. J BIOENERG BIOMEMBR. (2022) 54:155–62. doi: 10.1007/s10863-022-09940-9 35676565

[37] Li T Ji X Liu J Guo X Pang R Zhuang H. Ulinastatin improves renal microcirculation by protecting endothelial cells and inhibiting autophagy in a septic rat model. Kidney Blood Press Res (2022) 47:256–69. doi: 10.1159/000521648 35016182

[38] Howell GM Gomez H Collage RD Loughran P Zhang X Escobar DA. Augmenting autophagy to treat acute kidney injury during endotoxemia in mice. PloS One (2013) 8:e69520. doi: 10.1371/journal.pone.0069520 23936035 PMC3728340

[39] Karagiannidis I Kataki A Glustianou G Memos N Papalois A Alexakis N. EXTENDED CYTOPROTECTIVE EFFECT OF AUTOPHAGY IN THE LATE STAGES OF SEPSIS AND FLUCTUATIONS IN SIGNAL TRANSDUCTION PATHWAYS IN a RAT EXPERIMENTAL MODEL OF KIDNEY INJURY. SHOCK (2016) 45:139–47. doi: 10.1097/SHK.0000000000000505 26513702

[40] Li K Liu TX Li JF Ma YR Liu ML Wang YQ. rhEPO inhibited cell apoptosis to alleviate acute kidney injury in sepsis by AMPK/SIRT1 activated autophagy. Biochem Biophys Res Commun (2019) 517:557–65. doi: 10.1016/j.bbrc.2019.07.027 31383361

[41] Liu JX Yang C Zhang WH Su HY Liu ZJ Pan Q. Disturbance of mitochondrial dynamics and mitophagy in sepsis-induced acute kidney injury. Life Sci (2019) 235:116828. doi: 10.1016/j.lfs.2019.116828 31479679

[42] Liu JX Yang C Liu ZJ Su HY Zhang WH Pan Q. Protection of procyanidin B2 on mitochondrial dynamics in sepsis associated acute kidney injury via promoting Nrf2 nuclear translocation. Aging (Albany NY). (2020) 12:15638–55. doi: 10.18632/aging.103726 PMC7467384 32805725

[43] Yang T Feng X Zhao Y Zhang H Cui H Wei M. Dexmedetomidine enhances autophagy *via* alpha2-AR/AMPK/mTOR pathway to inhibit the activation of NLRP3 inflammasome and subsequently alleviates lipopolysaccharide-induced acute kidney injury. Front Pharmacol (2020) 11:790. doi: 10.3389/fphar.2020.00790 32670056 PMC7326938

[44] Zhao Y Feng X Li B Sha J Wang C Yang T. Dexmedetomidine protects against lipopolysaccharide-induced acute kidney injury by enhancing autophagy through inhibition of the PI3K/AKT/mTOR pathway. Front Pharmacol (2020) 11:128. doi: 10.3389/fphar.2020.00128 32158395 PMC7052304

[45] Yu YY Li XQ Hu WP Cu SC Dai JJ Gao YN. Self-developed NF-kappaB inhibitor 270 protects against LPS-induced acute kidney injury and lung injury through improving inflammation. BioMed PHARMACOTHER. (2022) 147:112615. doi: 10.1016/j.biopha.2022.112615 35026488

[46] Hsiao HW Tsai KL Wang LF Chen YH Chiang PC Chuang SM. The decline of autophagy contributes to proximal tubular dysfunction during sepsis. SHOCK (2012) 37:289–96. doi: 10.1097/SHK.0b013e318240b52a 22089196

[47] Mei S Livingston M Hao J Li L Mei C Dong Z. Autophagy is activated to protect against endotoxic acute kidney injury. Sci Rep (2016) 6:22171. doi: 10.1038/srep22171 26916346 PMC4768171

[48] Sunahara S Watanabe E Hatano M Swanson PE Oami T Fujimura L. Influence of autophagy on acute kidney injury in a murine cecal ligation and puncture sepsis model. Sci Rep (2018) 8:1050. doi: 10.1038/s41598-018-19350-w 29348412 PMC5773584

[49] Dai XG Xu W Li T Lu JY Yang Y Li Q. Involvement of phosphatase and tensin homolog-induced putative kinase 1-parkin-mediated mitophagy in septic acute kidney injury. Chin Med J (Engl). (2019) 132:2340–7. doi: 10.1097/CM9.0000000000000448 PMC6819035 31567378

[50] Feng LX Zhao F Liu Q Peng JC Duan XJ Yan P. Role of Nrf2 in lipopolysaccharide-induced acute kidney injury: protection by human umbilical cord blood mononuclear cells. Oxid Med Cell Longev (2020) 2020:6123459. doi: 10.1155/2020/6123459 32774680 PMC7407026

[51] Deng Z Sun M Wu J Fang H Cai S An S. SIRT1 attenuates sepsis-induced acute kidney injury *via* Beclin1 deacetylation-mediated autophagy activation. Cell Death Dis (2021) 12:217. doi: 10.1038/s41419-021-03508-y 33637691 PMC7910451

[52] Sun M Li J Mao L Wu J Deng Z He M. p53 deacetylation alleviates sepsis-induced acute kidney injury by promoting autophagy. Front Immunol (2021) 12:685523. doi: 10.3389/fimmu.2021.685523 34335587 PMC8318785

[53] Yang N Wang H Zhang L Lv J Niu Z Liu J. Long non-coding RNA SNHG14 aggravates LPS-induced acute kidney injury through regulating miR-495-3p/HIPK1. Acta Biochim Biophys Sin (Shanghai). (2021) 53:719–28. doi: 10.1093/abbs/gmab034 33856026

[54] Shintani T Klionsky DJ. Autophagy in health and disease: a double-edged sword. SCIENCE (2004) 306:990–5. doi: 10.1126/science.1099993 PMC1705980 15528435

[55] Vellai T Toth ML Kovacs AL. Janus-faced autophagy: a dual role of cellular self-eating in neurodegeneration? AUTOPHAGY (2007) 3:461–3. doi: 10.4161/auto.4282 17471017

[56] Chang KC Liu PF Chang CH Lin YC Chen YJ Shu CW. The interplay of autophagy and oxidative stress in the pathogenesis and therapy of retinal degenerative diseases. Cell Biosci (2022) 12:1. doi: 10.1186/s13578-021-00736-9 34980273 PMC8725349

[57] Kaushal GP Shah SV. Autophagy in acute kidney injury. Kidney Int (2016) 89:779–91. doi: 10.1016/j.kint.2015.11.021 PMC4801755 26924060

[58] Kimura T Takabatake Y Takahashi A Kaimori JY Matsui I Namba T. Autophagy protects the proximal tubule from degeneration and acute ischemic injury. J Am Soc NEPHROL. (2011) 22:902–13. doi: 10.1681/ASN.2010070705 PMC3083312 21493778

[59] Wang Z Huang Y Zhang J. Molecularly targeting the PI3K-Akt-mTOR pathway can sensitize cancer cells to radiotherapy and chemotherapy. Cell Mol Biol Lett (2014) 19:233–42. doi: 10.2478/s11658-014-0191-7 PMC6275747 24728800

[60] Xu G Mo L Wu C Shen X Dong H Yu L. The miR-15a-5p-XIST-CUL3 regulatory axis is important for sepsis-induced acute kidney injury. Ren Fail (2019) 41:955–66. doi: 10.1080/0886022X.2019.1669460 PMC6830252 31658856

[61] Li X Li J Zhang Y Zhou Y. Di-n-butyl phthalate induced hypospadias relates to autophagy in genital tubercle via the PI3K/Akt/mTOR pathway. J Occup Health (2017) 59:8–16. doi: 10.1539/joh.16-0089-OA 27885243 PMC5388616

[62] Dunlop EA Tee AR. mTOR and autophagy: a dynamic relationship governed by nutrients and energy. Semin Cell Dev Biol (2014) 36:121–9. doi: 10.1016/j.semcdb.2014.08.006 25158238

[63] Cui H Ren G Hu X Xu B Li Y Niu Z. Suppression of lncRNA GAS6-AS2 alleviates sepsis-related acute kidney injury through regulating the miR-136-5p/OXSR1 axis *in vitro* and in vivo. Ren Fail (2022) 44:1070–82. doi: 10.1080/0886022X.2022.2092001 PMC9272941 35793478

[64] Wang H Mou H Xu X Liu C Zhou G Gao B. LncRNA KCNQ1OT1 (potassium voltage-gated channel subfamily q member 1 opposite strand/antisense transcript 1) aggravates acute kidney injury by activating p38/NF-kappaB pathway *via* miR-212-3p/MAPK1 (mitogen-activated protein kinase 1) axis in sepsis. BIOENGINEERED (2021) 12:11353–68. doi: 10.1080/21655979.2021.2005987 PMC8810185 34783627

[65] Chen T Xue H Lin R Huang Z. MiR-34c and PlncRNA1 mediated the function of intestinal epithelial barrier by regulating tight junction proteins in inflammatory bowel disease. Biochem Biophys Res Commun (2017) 486:6–13. doi: 10.1016/j.bbrc.2017.01.115 28153728

[66] Palikaras K Lionaki E Tavernarakis N. Mechanisms of mitophagy in cellular homeostasis, physiology and pathology. Nat Cell Biol (2018) 20:1013–22. doi: 10.1038/s41556-018-0176-2 30154567

[67] Spoelstra-de MA Elbers P Oudemans-van SH. Making sense of early high-dose intravenous vitamin c in ischemia/reperfusion injury. Crit Care (2018) 22:70. doi: 10.1186/s13054-018-1996-y 29558975 PMC5861638

[68] Ma Y Qi M An Y Zhang L Yang R Doro DH. Autophagy controls mesenchymal stem cell properties and senescence during bone aging. Aging Cell (2018) 17:e12709. doi: 10.1111/acel.12709 29210174 PMC5770781

[69] Ng F Tang BL. Sirtuins' modulation of autophagy. J Cell Physiol (2013) 228:2262–70. doi: 10.1002/jcp.24399 23696314

[70] Martinez-Micaelo N Gonzalez-Abuin N Pinent M Ardevol A Blay M. Procyanidin B2 inhibits inflammasome-mediated IL-1beta production in lipopolysaccharide-stimulated macrophages. Mol Nutr Food Res (2015) 59:262–9. doi: 10.1002/mnfr.201400370 25379992

[71] Poon WH Ling RR Yang IX Luo H Kofidis T MacLaren G. Dexmedetomidine for adult cardiac surgery: a systematic review, meta-analysis and trial sequential analysis. ANAESTHESIA (2023) 78:371–380. doi: 10.1111/anae.15947 36535747

[72] Shi J Yu T Song K Du S He S Hu X. Dexmedetomidine ameliorates endotoxin-induced acute lung injury in vivo and in vitro by preserving mitochondrial dynamic equilibrium through the HIF-1a/HO-1 signaling pathway. Redox Biol (2021) 41:101954. doi: 10.1016/j.redox.2021.101954 33774474 PMC8027777

[73] Tang J Bassham DC. Autophagy during drought: function, regulation, and potential application. Plant J (2022) 109:390–401. doi: 10.1111/tpj.15481 34469611

[74] Wu Z Deng J Zhou H Tan W Lin L Yang J. Programmed cell death in sepsis associated acute kidney injury. Front Med (Lausanne). (2022) 9:883028. doi: 10.3389/fmed.2022.883028 35655858 PMC9152147

