# Supplementary figures and images for: Epigenetic dysregulation of autophagy in sepsis-induced acute kidney injury: the underlying mechanisms for renoprotection
Source: Front Immunol. 2023 May 5;14:1180866. doi: 10.3389/fimmu.2023.1180866 (PMC10196246; doi:10.3389/fimmu.2023.1180866)

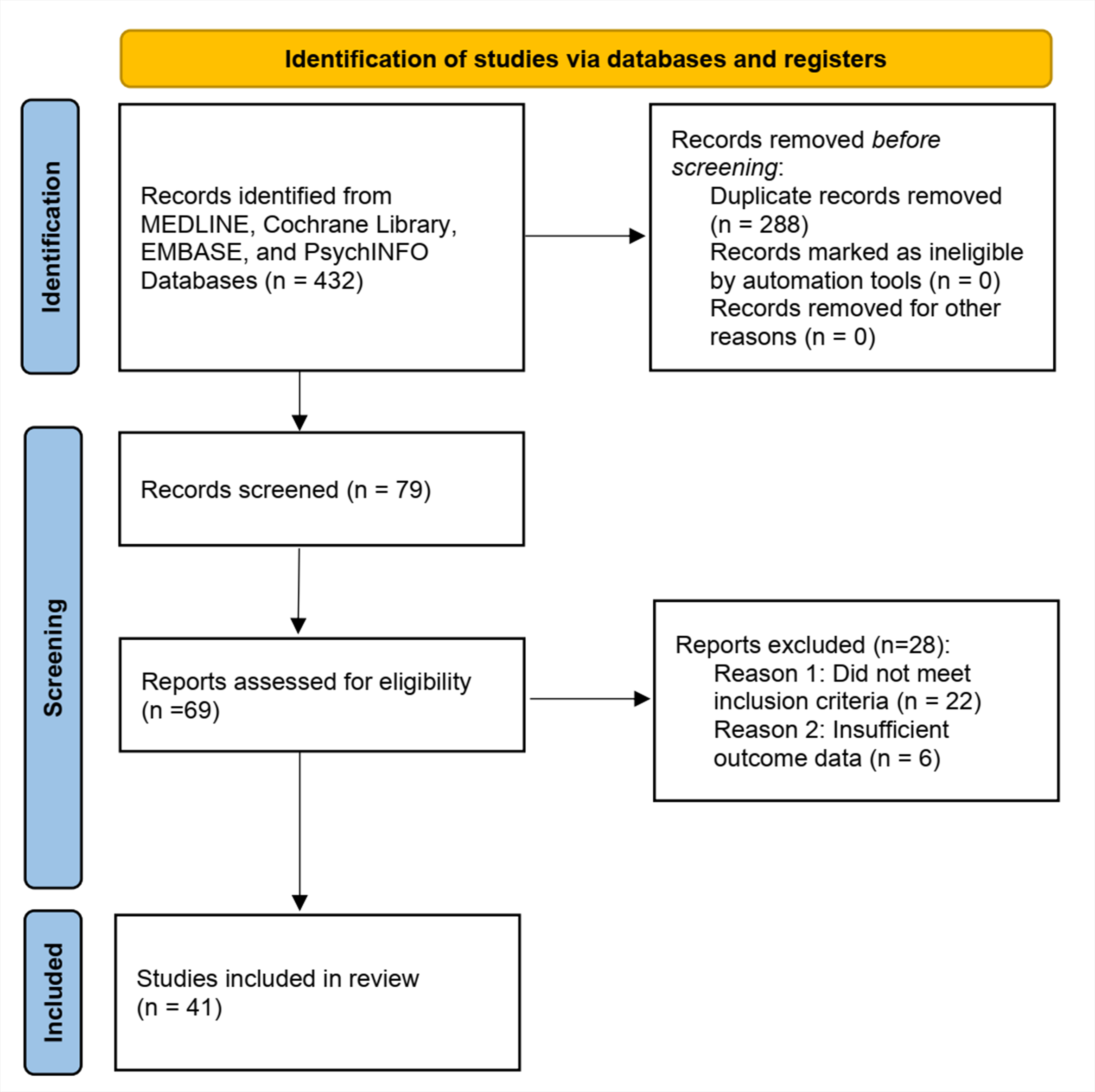

Supplement: Supplementary file 1 [file Image_1.tif]
