# Supplementary material for: Epigenetic dysregulation of autophagy in sepsis-induced acute kidney injury: the underlying mechanisms for renoprotection
Source: Front Immunol. 2023 May 5;14:1180866. doi: 10.3389/fimmu.2023.1180866 (PMC10196246; doi:10.3389/fimmu.2023.1180866)
Supplement: Supplementary file 2 [file Table_1.docx]

**Search strategy**

Through the use of MeSH and keywords, the search strategy in the MEDLINE database was followed: ((((((((((((((((((((((((("Acute Kidney Injury"[Mesh]) OR (Acute Kidney Injuries)) OR (Kidney Injuries, Acute)) OR (Kidney Injury, Acute)) OR (Acute Renal Injury)) OR (Acute Renal Injuries)) OR (Renal Injuries, Acute)) OR (Renal Injury, Acute)) OR (Renal Insufficiency, Acute)) OR (Acute Renal Insufficiencies)) OR (Renal Insufficiencies, Acute)) OR (Acute Renal Insufficiency)) OR (Kidney Insufficiency, Acute)) OR (Acute Kidney Insufficiencies)) OR (Kidney Insufficiencies, Acute)) OR (Acute Kidney Insufficiency)) OR (Kidney Failure, Acute)) OR (Acute Kidney Failures)) OR (Kidney Failures, Acute)) OR (Acute Renal Failure)) OR (Acute Renal Failures)) OR (Renal Failures, Acute)) OR (Renal Failure, Acute)) OR (Acute Kidney Failure)) AND (((((((((((((((((("Sepsis"[Mesh]) OR (Bloodstream Infection)) OR (Bloodstream Infections)) OR (Infection, Bloodstream)) OR (Pyemia)) OR (Pyemias)) OR (Pyohemia)) OR (Pyohemias)) OR (Pyaemia)) OR (Pyaemias)) OR (Septicemia)) OR (Septicemias)) OR (Poisoning, Blood)) OR (Blood Poisoning)) OR (Blood Poisonings)) OR (Poisonings, Blood)) OR (Severe Sepsis)) OR (Sepsis, Severe))) AND (((((((((("Autophagy"[Mesh]) OR (Autophagy, Cellular)) OR (Cellular Autophagy)) OR (Autophagocytosis)) OR (Reticulophagy)) OR (ER-Phagy)) OR (ER Phagy)) OR (Nucleophagy)) OR (Ribophagy)) OR (Lipophagy)).
